# Dental Works in Preparation for the Press

**Published:** 1852-10

**Authors:** 


*Dental Works in Preparation for the Press.*—We learn that Dr. W. H. Dwinelle is engaged in writing a history of American Dentistry, Biography &c., &c. A work upon these subjects from the pen of so competent a writer could scarcely fail to be interesting, and would be read by the members of the dental profession generally. The materials for a book of this sort are abundant. We also learn that Dr. D. has been engaged for two years past on a work on Microscopical Dental Anatomy, giving the appearance of the teeth in disease and in health, and also of the dental pulp through all its various manifestations, under the microscope. The work, we understand, will be copiously illustrated with engravings. We are glad to know that American dentists are directing their attention to researches of this kind, and that in the rivalry to excel in the practical departments, now so manifest in various parts of the country, scientific dentistry is not wholly lost sight of.

It also gives us pleasure to announce that Professor A. S. Piggot, M. D., of Baltimore, is writing a treatise on Dental Chemistry and Metallurgy, a work much needed; and we know of no one better qualified to prepare such a book than the above named gentleman. A book upon this subject would, we have no doubt, command a ready and extensive sale. The want of such a work has been long felt by the members of the dental profession and especially by the students of dental surgery.

